# Fall Injuries: An Important Preventable Cause of Trauma

**DOI:** 10.5812/atr.16079

**Published:** 2013-12-01

**Authors:** Mehrdad Mahdian

**Affiliations:** 1 Trauma Research Center, Kashan University of Medical Sciences, Kashan, IR Iran

**Keywords:** Fall, Injury, Trauma

Fall is one of the most common causes of major injuries and responsible for many hospital admissions in Iran (1, 2). Falling may happen at any age, but children and the aged people are at higher risk for it. Approximately 47000 children fall to their death annually, but hundreds of thousands more sustain less serious injuries from a fall (3). Falls represent the third leading cause of death in children and are responsible for 5.9% of childhood deaths due to trauma (4). Generally, death due to falls is resulted from head injury (5). Falls in children tend to be from terraces, tables, windows, and trees, and most frequently have a tendency to occur in homes, followed by playgrounds and schoolyards (6). Non-occupational falls from ladders and scaffolds have increasing incidence with increasing age. One-fifth of the cases result in hospitalization (7). Commonly, falls in the elderly occur during daily living activities. Older adults are five times more likely to be hospitalized due to falls than to injuries from other causes (8). Personal factors such as gait disturbance and muscle weakness, osteoarthritis, visual deficit, medications and cognitive impairment are the main causes of ground-level and stair falls, but such environmental elements as poor lighting and absence of handrails may enhance the frequency (4). In our country, Iran, injuries are responsible for most of YLD (Years Lived with Disability) in the population older than 80 years. This is most notably due to falls, and is often worsened by osteoporosis (9). Fall may occur in different places and for different reasons. In occupational settings, the most frequent type of accidents is fall from a height (10). In most cases (like ladder injuries), ignoring safety precautions result in fall. For example, fall from walnut trees is responsible for significant mortality and morbidity amongst those engaged in agricultural activities, and every year many victims of falling from walnut and almond trees are admitted to our trauma center during harvest season. The same can be seen in those involved in high altitude working activities e.g. construction workers. Almost in all cases some evidence of poor safety measures are observed. On the other hand, there are occasional cases of fall for such accidents as defective equipments in the entertainments parks.

Many of the fall cases occur at home setting. For example, during the weeks ending to Norouz (Iranian New Year) it is customary for the Iranians to do some special home cleaning activities which is known as Khaneh Takani. Indeed, women will be involved in cleaning every part of their home which are not routinely paid attention, and during this process falling scenarios are not uncommon. The head, spine, and extremities are the most commonly injured parts during fall. Unfortunately, among all these injuries, spinal injuries have the worst outcome. These injuries may cause life-long physical disabilities as well as consequential psychological problems like depression and impose a heavy burden to the patients and their family.

Fortunately, most of these accidents are preventable. Following these simple precautions may help preventing fall in children: using protective bars on windows, safety strap on highchairs and other infant seats, stair gates to prevent falling for infants and toddlers, keeping the maximum height of playgrounds equipment less than 1.5 - 2 meters, setting absorption level for a playground with sand or shredded rubber. Some environmental re-arrangements for hazard reduction with the use of handrails in bathrooms and stairs, monitoring and adjusting medications and providing appropriate light in living places may decrease fall frequency in older people. In occupational settings employers must organize a safe workplace and provide necessary information, instruction, equipments, training and supervision. Workers are also required to run the risk of fall prevention protocols such as using safe work systems, avoiding drug use before working at height and appropriate selection and use of personal protective equipments. The problem to achieve these measures is both social and personal acceptance and commitment for their application. Such an event does not seem to be accessible without solid legislative support. In the hope of having no more fall injuries in the new future.
